# The Evolution of Iron-Related Comorbidities and Hospitalization in Patients with Hemochromatosis: An Analysis of the Nationwide Inpatient Sample

**DOI:** 10.1097/BS9.0000000000000151

**Published:** 2023-01-17

**Authors:** Ahmad Abou Yassine, Kira MacDougall, Roula Sasso, Youssef Shammaa, Mira Alsheikh, Mohammad Abureesh, Loai Dahabra, Mohammad Alshami, Stephen Mulrooney

**Affiliations:** a Department of Internal Medicine, Zucker School of Medicine at Hofstra/Northwell at Staten Island University Hospital, New York, NY, USA; b Department of Gastroenterology, Zucker School of Medicine at Hofstra/Northwell at Staten Island University Hospital, New York, NY, USA

**Keywords:** Diabetes mellitus, Hemochromatosis, Hepatocellular carcinoma, Hospitalization, Iron overload, Liver cirrhosis

## Abstract

Hemochromatosis, either hereditary hemochromatosis (HH) or secondary hemochromatosis, consists of the accumulation of iron in the liver, heart, and other organs. It leads to end-organ damage in a proportion of affected subjects. Although liver-related morbidity (cirrhosis and hepatocellular carcinoma [HCC]) and mortality are well established, the frequency of these complications remains controversial. The aim of this study is to examine the rate of hospitalization and the incidence of iron overload-related comorbidities in patients with hemochromatosis between the years of 2002 and 2010. We queried the Nationwide Inpatient Sample (NIS) database from the year 2002 to 2010. We included adults (age ≥18 years) and used the ICD-CM 9 code 275.0x to identify hospitalized patients with a diagnosis of hemochromatosis. Data analysis for this study was generated using SAS software version 9.4. A total of 168,614 hospitalized patients between 2002 and 2010 had a diagnosis of hemochromatosis. The majority were males (57%) with a median age of 54 years (37–68), with a predominance of white patients (63.3%) followed by black (26.8%). The rate of hospitalization among patients with hemochromatosis increased by 79% between the years 2002 and 2010 (34.5/100,000 in 2002 vs 61.4/100,000 in 2010). The main associated diagnoses were diabetes mellitus (20.2%), cardiac disease, including arrhythmias (14%) and cardiomyopathy (dilated 3.8%; peri-, endo-, myocarditis 1.3%), liver cirrhosis (8.6%), HCC (1.6%), and acute liver failure (0.81%). Of note, HCC was associated with cirrhosis in 1188 patients (43% of HCC patients) and male sex (87%). Diagnostic biopsies were performed in 6023 (3.6%) of those patients and liver transplant was performed in 881 (0.5%). In-hospital mortality occurred in 3638 (2.16%) patients. In this large database study, we found a rising trend in hospitalization for hemochromatosis, possibly due to the increased recognition of this entity and billing for the condition. The incidence of cirrhosis in hemochromatosis was found to be similar to other studies (8.6% vs 9%). However, the rate of HCC was lower than previous reports (1.6% vs 2.2%–14.9%), and only 43% of HCC was associated with cirrhosis. This raises important pathophysiologic questions regarding the impact of iron overload in HCC. There has been an increase in the rate of hospitalization for patients with a diagnosis of hemochromatosis. This may be related to an increased recognition of hemochromatosis as the underlying etiology for conditions such as diabetes, cardiomyopathy, cirrhosis, and HCC. Further prospective studies are needed to clarify the burden of liver disease in HH and secondary iron overload.

## 1. INTRODUCTION

Hepatocellular carcinoma (HCC) is the third most fatal malignancy in the world.^1^ It is also one of the most common and its incidence is increasing.^2^ Regardless of etiology, the most important risk factor for HCC is cirrhosis.^3^ Cirrhosis may be caused by viral hepatitis (hepatitis B virus and hepatitis C virus), alcoholic liver disease, non-alcoholic fatty liver disease, autoimmune diseases, or hemochromatosis. Hereditary hemochromatosis (HH) was the first condition in which iron overload in the liver was shown to predispose patients to HCC. HH is characterized by increased absorption of dietary iron that results in the accumulation of iron in the liver, heart, and other organs. This leads to end-organ damage in a proportion of affected individuals.

Although the association between liver-related complications (cirrhosis and HCC) and mortality is well established, the frequency of these complications in patients with HH remains controversial. Most studies have reported an incidence of HCC of approximately 8%–10% in patients with HH,^4,5^ while other studies have demonstrated figures as low as 1.7%.^6^

Additionally, the prevalence of extra-hepatic complications of HH, such as diabetes mellitus, cardiomyopathy and cardiac arrhythmias among patients with HH varies widely within the literature. In this Study, we aim to examine the rate of hospitalization and the incidence of iron overload-related comorbidities, such as cirrhosis, HCC, diabetes mellitus, and cardiac diseases in patients with HH between the years of 2002 and 2010 in the United States.

## 2. METHODS

### 2.1. Data source

The Nationwide Inpatient Sample (NIS) was used to identify adult (≥18 years) patients with hemochromatosis over a 9-year period (2002-2010). Diagnosis codes were used to identify patients with hemochromatosis using ICD-CM 9 code 275.0x, as well as other ICD-CM 9 codes for comorbidities of interest (Table 1).

**Table 1:** Diagnosis and respective ICD-9 codes.

Diagnosis	ICD code
Hemochromatosis	275.0x
Diabetes mellitus with and without complications	24900 24901 24910 24911 24920 24921 24930 24931 24940 24941 24950 24951 24960 24961 24970 24971 24980 24981 24990 24991 25000-25003 25010-25013 25020-25023 25030-25033 25040-25043 25050-25053 25060-25063 25070-25073 25080-25083 25090-25093 7902 7915 7916 79021 79022 79029 V4585 V5391 V6546
Peri-; endo-; and myocarditis; cardiomyopathy (except that caused by tuberculosis or sexually transmitted disease)	03282 03640-03643 07420-07423 11281 11503 11504 11513 11514 11593 11594 1303 393 3910-3912 3918-3920 3980 39890 39899 4200 42090 42091 42099 4210 4211 4219 4220 42290-42293 42299 4230-4233 4238-4251 42511 42518 4252-4254-4290
Arrythmia	4270 4272 4279 7850 7851 42731 42732 42760 42761 42769 42781 42789
Acute liver failure	570
Cirrhosis	5715
Transplant	V427
Hepatocellular carcinoma	155.x
Dilated cardiomyopathy	4254

Data analysis for this study was generated using SAS software version 9.4 using the SURVEY procedures. Categorical variables were presented as numbers and percentages; and continuous variables as median with interquartile ratio (IQR). Univariate analysis was conducted to estimate the effect of factors that affected mortality and multivariate analysis models for mortality included variables that reached statistical significance at *P* value <.05.

Given that the data from NIS is de-identified, IRB approval was waived.

## 3. RESULTS

**Figure 1.**
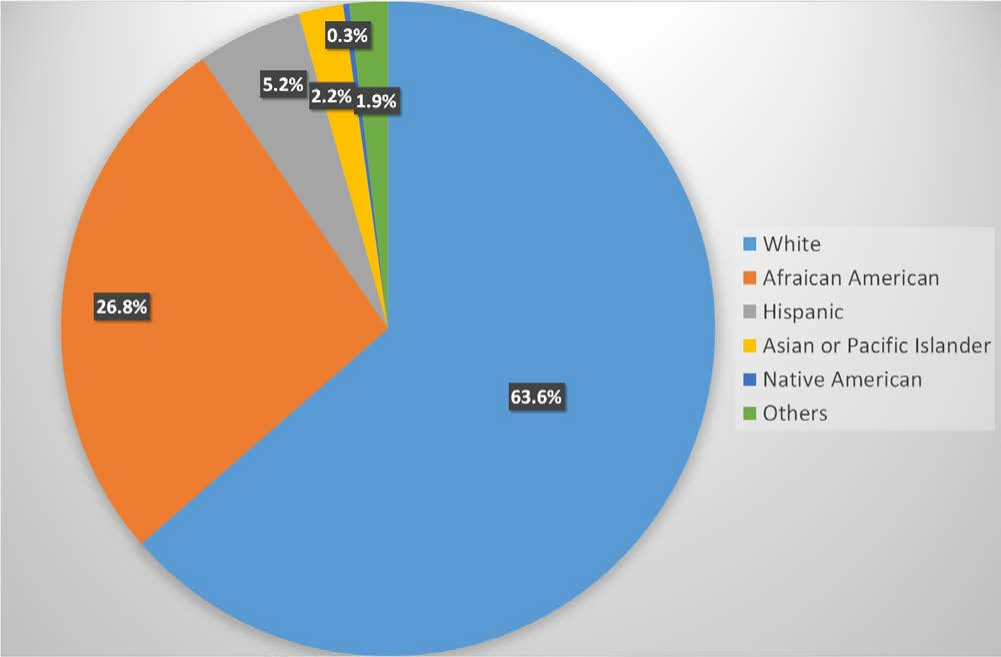
Prevalence of hemochromatosis in hospitalized patients in the United States by race.

A total of 168,614 hospitalized patients between 2002 and 2010 with a diagnosis of hemochromatosis were identified. Baseline characteristics and patient clinical outcomes can be seen in Table 2. Most patients were male (57%) and Caucasian (63.3%), and the median age was 54 years (IQR 37–68) (Figure 1). Among these patients, the most common comorbidity of interest was diabetes mellitus, occurring in 20% of the patients, followed by cardiac arrhythmias (14%) and cirrhosis (8.6%). Hepatocellular carcinoma occurred in 1.6% of the patients. The median length of hospital stay was 4 days (IQR 2–7 days) and median hospital cost was $189,000 (98,000–368,000). In-hospital mortality occurred in 3638 (2.16%) patients (Table 2).

**Table 2:** Demographic characteristic and outcomes in hospitalized patients with hemochromatosis.

Demographics	N (%)
Total	168,614
Male	96,110 (57)
Age (median, IQR)	54 (37–68)
Age <40	49,419 (29.3)
Age 40–60	54,797 (32.5)
Age >60	64,162 (38.1)
Race	
White	87,149 (63.6)
Black	36,736 (26.8)
Hispanic	7149 (5.2)
Asian or Pacific Islander	3029 (2.2)
Native American	429 (0.3)
Other	2533 (1.9)
Diabetes mellitus	34,085 (20.2)
Arrythmia	23,600 (14)
Cirrhosis	15,545 (8.63)
Dilated cardiomyopathy	6427 (3.8)
HCC and cholangiocarcinoma	2704 (1.6)
Cardiomyopathy peri-, endo-, myocarditis	2086 (1.3)
Acute liver failure	1370 (0.81)
Cirrhosis and HCC	1188 (0.7)
Transplant	881 (0.5)
Liver biopsy	6023 (3.6)
Cost (1000 USD)	18.7 (9.8–36.8)
Length of stay, days (median, IQR)	4 (2–7)
Mortality	3,638 (2.16)

HCC = hepatocellular carcinoma, IQR = interquartile ratio.

When evaluating the comorbidities associated with hemochromatosis, stratified by sex (Table 3), results demonstrated males were predisposed to develop HH-related complications compared to females.

**Table 3:** Comorbidities and outcomes stratified by patient sex.

Variable	Total population (%), Total = 168,518	Male, N = 96,072 (57)	Female, N = 72,445 (43)	OR (95% CI)
Diabetes mellitus	34,085 (20.2)	22,420 (23.3)	11,665 (16.1)	1.58 (1.54-1.62)
Arrhythmias	23,594 (14)	14,579 (15.2)	9015 (12.4)	1.25 (1.22-1.29)
Cirrhosis	14,540 (8.6)	9792 (10.2)	4748 (6.6)	1.61 (1.56-1.67)
Cardiomyopathy	8513 (5.1)	5502 (5.7)	3011 (4.2)	1.40 (1.34-1.47)
Dilated CM	6426 (3.8)	4199 (4.4)	2227 (3.1)	1.44 (1.36-1.51)
Deceased	3638 (2.2)	2258 (2.4)	1380 (1.9)	1.23 (1.15-1.32)
Hepatocellular carcinoma and/or cholangiocarcinoma	2704 (1.6)	2359 (2.5)	345 (0.5)	5.25 (4.69-5.88)
Combined hepatocellular carcinoma and/or cholangiocarcinoma; and cirrhosis	1188 (0.7)	1013 (1.1)	175 (0.2)	4.41 (3.75-5.18)
Transplant	880 (0.5)	679 (0.7)	201 (0.3)	2.55 (2.18-2.99)

CI = confidence interval, OR = odds ratio.

We additionally conducted univariate and multivariate analysis to identify the independent predictors of mortality in patients with hemochromatosis (Table 4) and found the following variables to predictors of mortality in both the univariate and multivariate analysis: age, cirrhosis, malignancy, development of acute liver failure, presence of cardiomyopathy/dilated cardiomyopathy, and cardiac arrhythmias (Table 4).

**Table 4:** Univariate and multivariate analysis for in-hospital mortality for patients with hemochromatosis.

Variable	UOR (95% CI)	*P* value	AOR (95% CI)	*P* value
Age (reference 0–40)	–	–	–	–
Age 40–60	2.1 (1.63-2.71)	<.0001	1.75 (1.35-2.26)	<.0001
Age 60–100	4.22 (3.3-5.39)	<.0001	3.59 (2.78-4.65)	<.0001
Sex (Male vs Female)	1.24 (1.06-1.45)	.0071	1.07 (0.91-1.26)	.4362
Acute liver failure	14.63 (10.94-19.57)	<.0001	16.88 (12.24-23.28)	<.0001
Malignancy	4.95 (3.63-6.74)	<.0001	2.68 (1.88-3.82)	<.0001
Cirrhosis	3.24 (2.7-3.89)	<.0001	2.67 (2.17-3.29)	<.0001
Cardiomyopathy	2.27 (1.78-2.9)	<.0001	3.63 (2.42-5.43)	<.0001
Arrhythmias	2.17 (1.83-2.58)	<.0001	1.61 (1.34-1.93)	<.0001
Dilated cardiomyopathy	1.78 (1.31-2.41)	<.0001	0.42 (0.25-0.69)	.0006
Diabetes	1.27 (1.07-1.51)	.0052	0.88 (0.74-1.06)	.1769

AOR = adjusted odds ratio, CI = confidence interval, UOR = unadjusted odds ratio.

**Figure 2.**
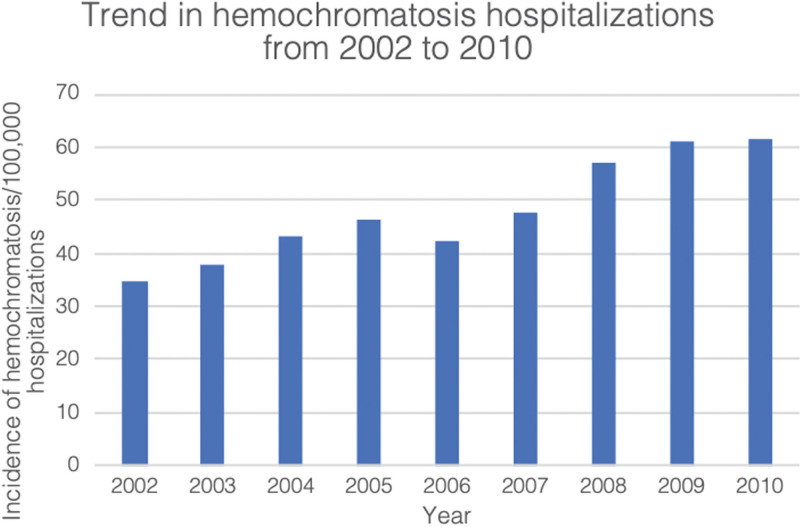
Trend in hemochromatosis hospitalizations incidence per 100,000 hospitalizations from 2002 to 2010.

The rate of hospitalization among patients with hemochromatosis increased by 79% between the years 2002 and 2010 (34.5/100,000 in 2002 vs 61.4/100,000 in 2010), demonstrated in Figure 2.

## 4. DISCUSSION

This study analyzed data from 168,614 hospitalized patients in the United States with a diagnosis of hemochromatosis between the years 2002 and 2010 and identified the prevalence of complications associated with hemochromatosis in this subgroup of patients. To our knowledge, this is the largest database study of its kind performed on patients with HH in the United States. Our study showed that diabetes mellitus was the most commonly associated comorbidity in this subgroup of patients and mortality was 2.2%.

A rising hospitalization trend for HH from 44/100,000 to 61/100,000 was noted from 2002 to 2010 Figure 2. Also, this study showed that the prevalence of HH was 47.6/100,000 compared to 412 per 100,000 in a Norwegian survey conducted in white non-Hispanic population.^7^HH is known to affect Caucasian and North European descents group more than others,^8^ which can explain the lower prevalence found in our study.

The prevalence of medical conditions associated with HH found in our study were consistent with those previously reported in the literature. A patient registry in Southern France found the prevalence of cirrhosis among this population to be 9%,^9^ which is consistent with our reported 8.6%.

HCC can develop in non-cirrhotic patients with HH as well, suggesting that iron may play a role in liver carcinogenesis independent of cirrhosis. This hypothesis is supported by a study of Borgna-Pignatti et al which demonstrated that patients with beta-thalassemia and iron overload secondary to transfusions, are also at an increased risk of developing HCC.^10^

Additionally, some studies have shown the prevalence of HCC among patients with hemochromatosis to be 2.2%, while other studies have demonstrated a higher prevalence of 14.9%, which is inconsistent with the prevalence of 1.6% obtained in our study. This could be due to the geographic and ethnic diversity between different studies.

Interestingly, of the patients in our study diagnosed with HCC, 87% of them were male and only 43% of them had a diagnosis of cirrhosis. In their study, Hafaeid et al found an association between male gender and HCC in the United States with an male-to-female ratio of 4:1.^11^ This can be explained by the protective role of estrogen in females and the higher incidence of hepatitis B virus infection among men.^12^ This raises important pathophysiologic questions regarding the impact of iron overload in HCC. Iron could be implicated in the development of HCC in patients with HH irrespective of its role in the development of cirrhosis. Iron may act as a carcinogen through mechanisms such as oxidative stress, facilitation of tumor growth, and modification of the immune system.^13^

In Addition, experimental studies support a carcinogenic or co-carcinogenic role of iron in the development of malignancies such as HCC and cholangiocarcinoma. Deugnier et al biopsied the livers of 185 patients with HH and identified a proliferative lesion called iron-free-foci, which are sublobular nodules of hepatocytes free of iron or exhibiting much less iron that the surrounding parenchyma.^14^ They prospectively followed these patients and found that 50% of the patients with iron-free-foci developed HCC compared to 8% from the control group.^14^ Their study demonstrated that iron-free-foci are a preneoplastic condition and places these patients at risk for the development of HCC. This highlights the importance of regular screening for HCC in patients with HH.

Even less is known about the incidence of cholangiocarcinoma in patients with HH and the role of iron overload in the development of this condition. Our study could not discern the isolated incidence of cholangiocarcinoma due to the absence of a single diagnostic code for cholangiocarcinoma. One study conducted by Morcos et al analyzed the livers of 20 patients with primary liver cancer in the background of HH. Histologically, 65% of the cases were HCC, 15% were cholangiocarcinoma, and 20% were hepatocholangiocarcinoma. They found that these malignancies could arise in non-cirrhotic livers and were often associated with Von Meyenburg Complexes (VMC), or biliary micro-hamartomas. This further highlights the importance of routine screening for primary liver cancer in patients with HH, especially if VMCs are identified.

Primary liver cancer is a major cause of death in patients with HH. Previous studies have shown that primary liver cancer accounts for approximately 27.5%–45% of deaths of patients with HH.^15,16^ Risk factors include male sex, age >50 years, cirrhosis, alcoholism, tobacco, and hepatitis B and C. One cohort study of 251 patients with HH found that the prognosis of HH and most of its complications, including HCC were dependent on the amount and duration of iron excess.^15^ The study concluded that early diagnosis and therapy could prevent the adverse consequences of iron overload.

We acknowledge limitation to this study, including limitations that are inherent to the NIS database such as its retrospective nature, lack of access to the methodology of the diagnosis of hemochromatosis, such as pathology or genetic testing. Additionally, this database only contains on data on hospitalized patients with hemochromatosis, and therefore, our study sample may represent more severe cases of patients with hemochromatosis. The NIS database does not identify readmissions. Considering that patients with hemochromatosis may have multiorgan disorders, readmissions are likely and thus resulting in overestimation of hospitalized patients with hemochromatosis.

## 5. Conclusion

There has been an increase in the rate of hospitalization for patients with a diagnosis of hemochromatosis. This may be related to an increased recognition of hemochromatosis as the underlying etiology for conditions such as diabetes mellitus, cardiomyopathies, liver cirrhosis, and HCC. Further prospective studies are needed to clarify the burden of liver disease in HH and secondary iron overload.
